# First person – Éva Saskői

**DOI:** 10.1242/dmm.047407

**Published:** 2020-10-15

## Abstract

First Person is a series of interviews with the first authors of a selection of papers published in Disease Models & Mechanisms, helping early-career researchers promote themselves alongside their papers. Éva Saskői is first author on ‘The SDHB Arg230His mutation causing familial paraganglioma alters glycolysis in a new *Caenorhabditis elegans* model’, published in DMM. Éva is a PhD student in the lab of Krisztina Takács-Vellai at Eötvös Lorand University, Budapest, Hungary, investigating the developmental functions of tumor suppressor homologs in *Caenorhabditis elegans*.


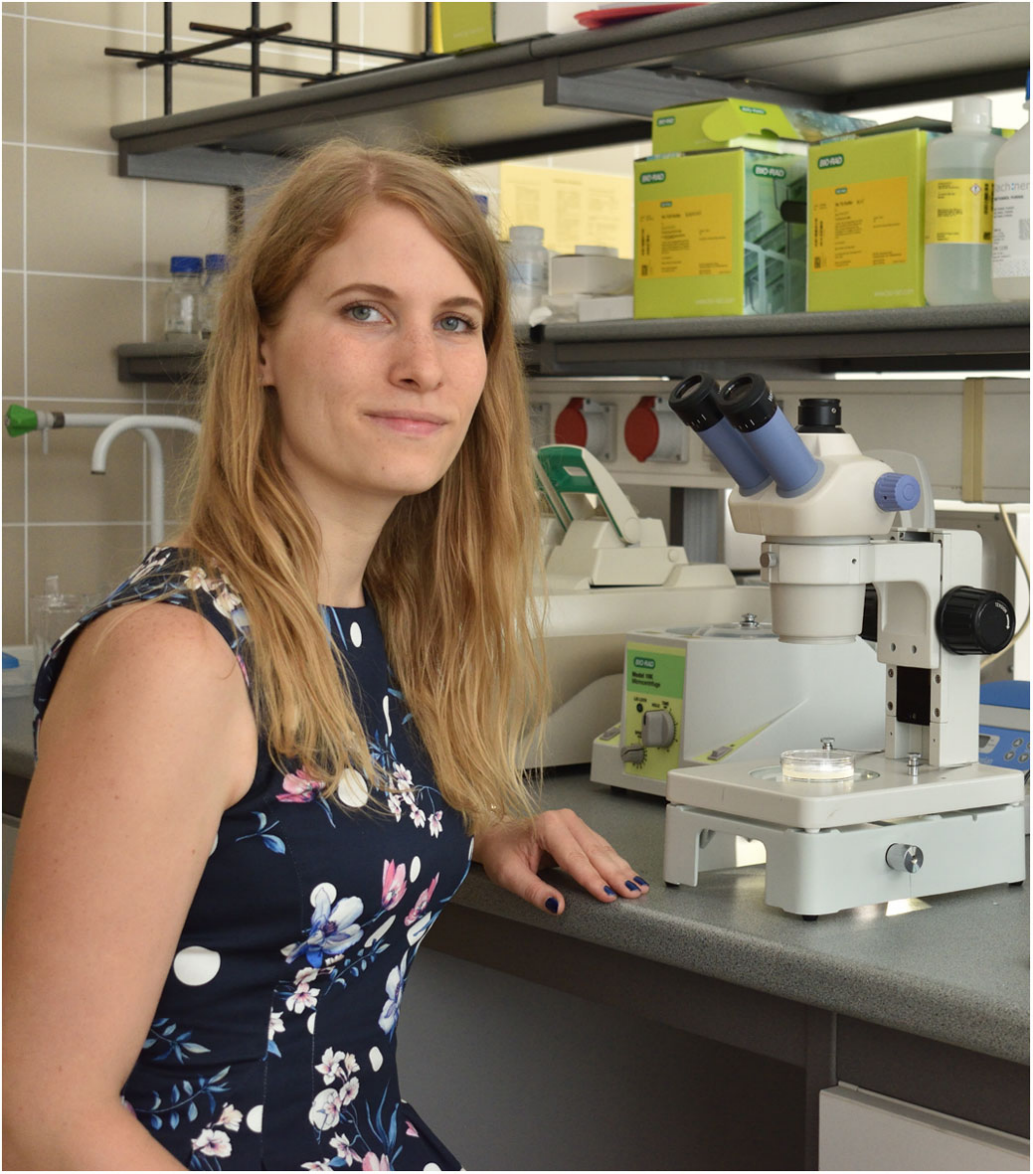


**Éva Saskői**

**How would you explain the main findings of your paper to non-scientific family and friends?**

Pheochromocytomas and paragangliomas are rare, inherited neuroendocrine tumors, which are now regrouped as pheochromocytoma/paraganglioma (PPGL) based on their histological and genetic features. They differ only in anatomical site of origin: pheochromocytoma is adrenal, while paraganglioma is a cancer of the sympathetic and parasympathetic ganglia of the peripheral nervous system. The annual PPGL incidence has been estimated at 2-8 per million people per year in the world, but, owing to the non-specificity of the presenting symptoms, which include arterial hypertension, sweating, headache and tachycardia, the condition is almost certainly underdiagnosed. Several mutant genes have been associated with PPGL, but the patient with heritable/germ line SDHB mutations (mutations in the gene encoding the B subunit of succinate dehydrogenase) suffers from a particularly aggressive form of malignant PPGL. The mechanisms behind are obscure; in addition, current treatment options for PPGL patients are limited.

To better understand the consequences of SDHB mutations in PPGLs, we developed an animal model by generating a clinically relevant mutation in the orthologous highly conserved *Caenorhabditis elegans* gene *sdhb-1*. We found that – compared to control worms – the development of mutant animals is facilitated by high lactate levels, a feature also relevant to tumor cells that have undergone metabolic reprogramming (called Warburg effect). We used a lactate dehydrogenase inhibitor, which selectively delayed the development of mutant worms while leaving normal worms unscathed, meaning that our model is druggable. Therefore, we believe that this *C. elegans* worm model could be used to test the effects of different pre-clinical drug candidates against PPGL tumors.

**What are the potential implications of these results for your field of research?**

The most outstanding result of our work is the detection of high lactate dehydrogenase activity of the *sdhb-1* mutant animals carrying the clinically relevant human mutation. Similar to these mutants, the majority of malignant tumor cells use elevated glycolysis to provide energy for cell proliferation and produce high lactate levels, which induce angiogenesis or inhibit the immune response. Vascular supply of tumors and the suppression of the immune system are key steps in metastasis formation. Therefore, our model can help to explain, and further investigate, why SDHB mutations are associated with high malignant potential. Because we can selectively treat the *sdhb-1* mutant animals with lactate dehydrogenase inhibitor, we think that our novel *C. elegans* model can serve as a pharmacological model of succinate dehydrogenase loss disorders, which might be used after optimization for future high-throughput screening of drug candidates.

“[…] 40% of genes associated with human diseases have a *C. elegans* ortholog, so many of the discoveries made in the nematode model can be considered relevant to the study of human health and disease.”

**What are the main advantages and drawbacks of the model system you have used as it relates to the disease you are investigating?**

I believe the most important advantage of using *C. elegans* is that 40% of genes associated with human diseases have a *C. elegans* ortholog, so many of the discoveries made in the nematode model can be considered relevant to the study of human health and disease. Many human genes whose mutations promote or inhibit tumorigenesis show a high degree of conservation in the nematode; therefore, this model is excellent for use in cancer research. The invariant somatic cell lineage of the worm model has been fully explored due to its transparent body; thus, phenotypes showing a pattern other than normal cell division can be easily identified. The nematodes contain mammalian homologs of key enzymes of glycolysis, gluconeogenesis, citric acid cycle and oxidative phosphorylation, so this model system is excellent for studying metabolic pathways as well. The limitation of using *C. elegans* as a cancer model is that adults of these organisms are mostly composed of post-mitotic cells; these somatic cells no longer undergo the cell cycle and therefore cannot develop cancer. Thus, the only tissue that develops tumorous cells is the germline.

**Figure:**
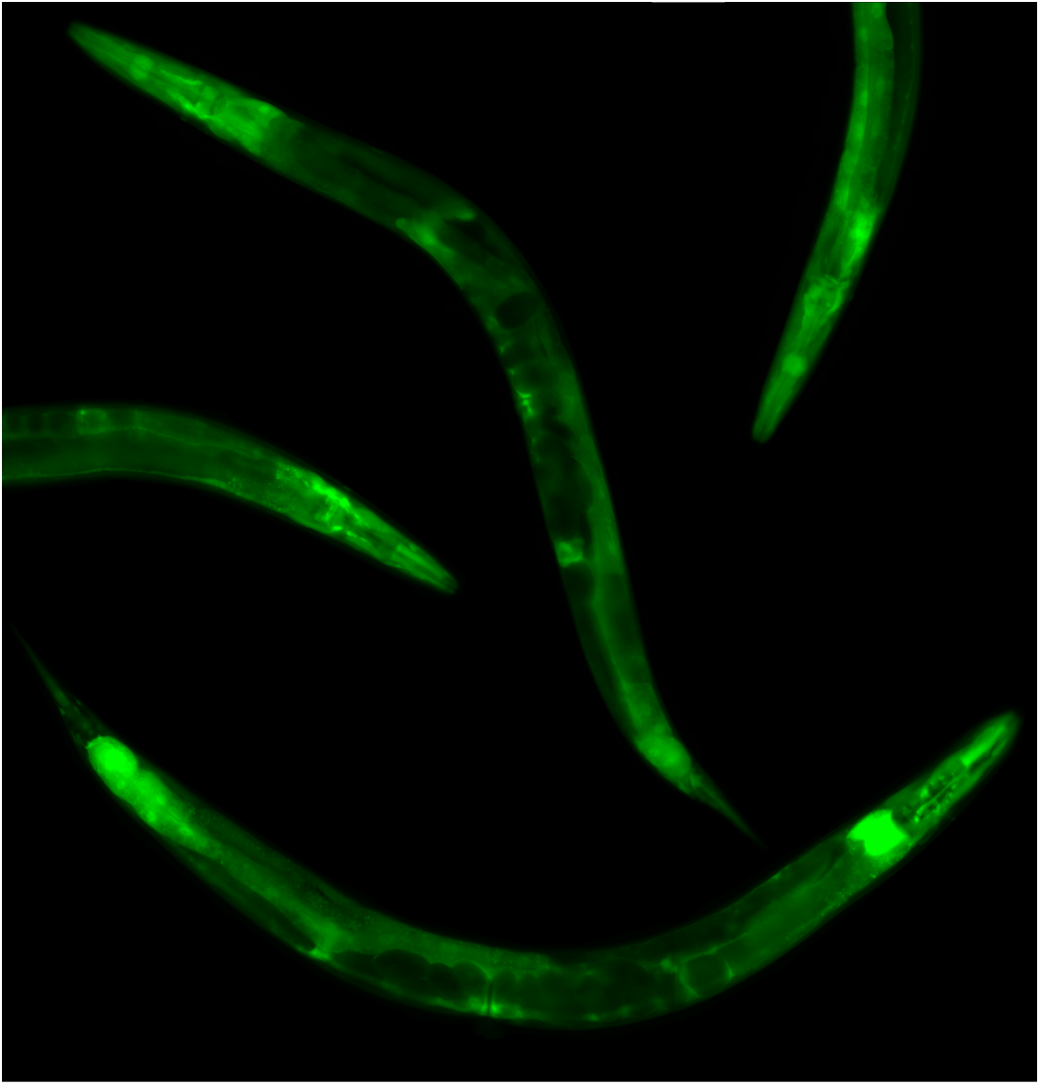
**Transgenic worms carrying GFP-tagged transcriptional *sdhb-1* show ubiquitous expression, but primarily in the nervous system.**

**What has surprised you the most while conducting your research?**

The biggest surprise to me was the high lactate levels and lactate dehydrogenase activity of *sdhb-1* mutant animals: a common feature of our mutants and tumor cells that have undergone metabolic reprogramming, meaning that our worms can indeed model altered energy metabolism (Warburg effect) of tumor cells.

**Describe what you think is the most significant challenge impacting your research at this time and how will this be addressed over the next 10 years?**

Since SDHB mutations cause cancer of neuroendocrine cells, the significant challenge impacting our research is to analyze metabolism of the four uv1 neuroendocrine cells in *sdhb-1* mutants and control animals. First, by using fluorescent markers labeling these cells specifically, it is important to see whether they are present and normally developed in our mutants. Next, it is possible to select the fluorescent neuroendocrine cells by fluorescence-activated cell sorting after chemical and mechanical disruption of the mutant worms. Over the next 10 years, liquid chromatography and mass spectrometry techniques will hopefully be developed so that a smaller amount of cells would be sufficient and easily analyzed.

**What changes do you think could improve the professional lives of early-career scientists?**

First, I believe it is very important for early-career scientists to find a supervisor who can motivate them, because that is the only way to produce results. I believe that early-career scientists would benefit from more opportunities to receive scholarships and grants to help them implement their own innovative ideas as well. I consider collaborations to be very important too, and, if possible, scientists should visit other labs to learn new, modern technologies, which can be later useful in their own project.

**What's next for you?**

Mutant animals also show a significant accumulation of succinate, which promotes the activation of the hypoxic response by inhibiting the enzyme prolyl hydroxylase as an oncometabolite. Hypoxia is often observed inside tumors, where there is no longer sufficient oxygen for tumor cells, so understanding this mechanism in *sdhb-1* mutant animals is essential. We also want to use our model for drug testing to explore new treatment and therapeutic options for PPGL patients.

## References

[DMM047407C1] Saskői É., Hujber Z., Nyírő G., Likó I., Mátyási B., Petővári G., Mészáros K., Kovács A. L., Patthy L., Supekar S. et al. (2020). The SDHB Arg230His mutation causing familial paraganglioma alters glycolysis in a new *Caenorhabditis elegans* model. *Dis. Model. Mech.* 13, dmm044925 10.1242/dmm.044925 PMC7578352 32859697

